# Common Autoimmune Antibodies in Unexplained Infertile Female Patients in Saudi Arabia

**DOI:** 10.7759/cureus.31724

**Published:** 2022-11-21

**Authors:** Emad Koshak, Ali Atwah, Rajeh Aljedani, Yasser Aljaied, Mahmoud A Gaddoury

**Affiliations:** 1 Internal Medicine, Faculty of Medicine, King Abdulaziz University, Jeddah, SAU; 2 Pediatrics, Faculty of Medicine, King Abdulaziz University, Jeddah, SAU; 3 Radiology/Family Medicine, Faculty of Medicine, King Abdulaziz University, Jeddah, SAU; 4 Community Medicine, Faculty of Medicine, King Abdulaziz University, Jeddah, SAU

**Keywords:** antithyroid antibodies, antiphospholipid antibody, saudi arabia, unexplained infertility, autoimmune antibodies

## Abstract

Background: Unexplained female infertility is a devastating health problem that has become increasingly prevalent worldwide with no specific explanation despite comprehensive evaluations. Recent data suggest that serum autoimmune antibodies are frequently found in patients with unexplained female infertility.

Objectives: This study aims to identify the prevalence of common autoantibody abnormalities in females with unexplained infertility in Saudi Arabia.

Methods: A cross-sectional study was conducted on female patients with unexplained infertility sequentially referred to the allergy and immunology clinic at King Abdulaziz University Hospital (KAUH). Demographics, clinical characteristics, and 12 common autoantibody immunological tests were described as frequency and percentage. The chi-square test was applied to evaluate any associations.

Results: A total of 119 females with unexplained infertility were studied; of those, 97 (81.5%) had secondary infertility. Their average age was 33.9 ± 5.6 years ranging from 23 to 49 years. The overall prevalence of a high level of at least one autoantibody (above the normal range) was 84%. The predominant high autoantibodies were antithyroglobulin in 42 (35.3%), antithyroid microsomal in 41 (34.5%), beta 2 glycoprotein IgM in 40 (33.6%), antigliadin IgA in 32 (26.9%), antinuclear in 22 (18.5%), and anticardiolipin IgM in 18 (15.1%) of the participants. The following significant associations were found in our study: secondary infertility with beta 2 glycoprotein (p = 0.022), age with antithyroglobulin (p = 0.027), and the number of pregnancies with anti-tissue transglutaminase IgG and antigliadin IgA (p = 0.015 and p = 0.043, respectively).

Conclusion: A high level of at least one autoantibody was detected in the majority of females with unexplained infertility. Antithyroid, antiphospholipid, antigliadin, and antinuclear autoantibodies were the most common autoantibodies. These findings may encourage autoantibody screening in infertile females to discover any potential immunopathology in further clinical studies.

## Introduction

Infertility is defined as the failure to conceive after 12 months or more of regular unprotected sexual intercourse, and it has a global average prevalence of approximately 15% [1]. In Saudi Arabia, the infertility rates among women are on the rise and are currently up to 18.9-23.3% [2,3].

The most devastating type of infertility, which is also on the rise worldwide, is unexplained infertility, which accounts for about 10-17% of infertile females [4,5]. The dilemma is the absence of a clear reason for infertility despite thorough evaluations and treatments [5,6]. The approach to treatment is constantly changing as new evidence describes different possible etiologies with clinical associations, indicating newer laboratory investigations [5,6].

It has been well established that medical disorders associated with autoimmune antibodies, such as systemic lupus erythematosus and antiphospholipid syndrome, account for some of the infertility cases [5]. Over the last 20 years, nonspecific autoimmunity, defined as the presence of autoantibodies in a serum sample without the clinical criteria of a defined disease, has been described in some infertile women [7].

A recent systematic review has shown that serum autoantibodies, including antithyroid, antiphospholipid, and antinuclear antibodies, were more frequently found in females with unexplained infertility [7]. The presence of antinuclear antibodies is regarded as a typical feature of autoimmunity in recurrent pregnancy loss [8]. Moreover, antisperm antibodies as well as celiac disease antibodies have been linked to unexplained infertility cases [9,10]. Many studies have been conducted to clarify the association between these autoantibodies with not only recurrent abortions but also with repeated IVF failures, but the findings have been debatable [7,11].

According to many international guidelines, it is not routinely recommended to conduct immunological investigations in the approach to female infertility [12,13]. Nevertheless, the American and European societies for reproduction recommend screening for antiphospholipid antibodies only after two pregnancy losses, while many European societies of obstetricians and gynecologists recommend this investigation after three pregnancy losses [14]. More recently, some of these guidelines recommend screening for antithyroid antibodies and antinuclear antibodies in recurrent pregnancy losses for exploratory purposes [15]. Moreover, some authors have recently recommended ordering some autoimmune test workups after two in vitro fertilization (IVF) failures [11].

However, immunological investigations in females with unexplained infertility are rarely considered in infertility clinics, despite the possibility of silent immunological irregularities that might be easily underestimated by many physicians. Hence, this project was intended to explore the prevalence of any potential common auto-immunological laboratory abnormalities in females with unexplained infertility in Saudi Arabia.

## Materials and methods

Study design and setting

This is a cross-sectional study of female patients diagnosed with unexplained infertility at King Abdulaziz University Hospital (KAUH), Jeddah, Saudi Arabia. It was conducted from May to August 2022. KAUH is an 800-bed hospital, one of the tertiary referral and teaching centers in the western region of Saudi Arabia.

The inclusion criteria for the participants specified all females aged 18 to 45 years old with unexplained primary and secondary infertility sequentially referred to the allergy and immunology clinic at KAUH by infertility physicians to rule out immunological abnormalities. According to the WHO, primary infertility refers to females who have never fallen pregnant, while secondary infertility refers to females who have been pregnant once [1].

The exclusion criteria specified infertility cases with identified well-known causes other than abnormal immunological tests and with incomplete immunological laboratory results and missing follow-up.

The sample size and sampling procedures

A total of 136 female patients with unexplained infertility referred from many infertility specialists were recruited at KAUH. Of these, 17 were excluded according to our inclusion and exclusion criteria.

Data collection

The patient’s demographic, clinical, and laboratory data were collected through electronic medical record files. Data distribution and collection were conducted with the use of electronic Google Sheets (Google, Mountain View, CA) divided into three sections. The first section collected the patient’s demographic data. The second section consisted of eight points of clinical information, namely, infertility type, number of pregnancies, living children, preterm labor, abortions, stillbirths, intracytoplasmic sperm injection (ICSI), and intrauterine insemination (IUI). The third section was related to 12 common autoantibody immunological tests, and the autoantibodies were beta 2 glycoprotein antibody IgG, beta 2 glycoprotein antibody IgM, anticardiolipin antibody IgG, anticardiolipin antibody IgM, antithyroglobulin antibody, antithyroid microsomal antibody, antinuclear antibodies, antisperm antibody, anti-tissue transglutaminase antibody IgA, anti-tissue transglutaminase antibody IgG, antigliadin antibody IgA, and antigliadin antibody IgG. Testing for autoantibodies in the serum of the studied patients was performed at the immunology laboratory at KAUH.

Data analysis

The statistical analysis consisted of two parts. First, a descriptive analysis of all the subjects included in the study was conducted. Frequency and percentage were calculated for categorical factors, while means and standard deviation were calculated for continuous variables. Thereafter, the association between autoantibody laboratory tests and the sociodemographic characteristics and the obstetrics gynecological background variables were estimated using the chi-square test. All p-values < 0.05 were considered statistically significant. The Statistical Package for Social Sciences (SPSS) version number 26 (IBM Corp., Armonk, NY) was used for all data analyses.

Research ethics

The Research Committee of the Unit of Biomedical Ethics of KAUH approved the study proposal (reference number: 331-22). Participation was voluntary, participants were informed about the objectives and methodology of the study, and they were not offered any incentives to participate. Verbal consent was obtained from each participant before the data collection.

## Results

A total of 136 female patients with unexplained infertility referred from many infertility specialists were recruited at KAUH. Of these, 17 were excluded: 10 due to a lack of follow-up and seven for insufficient immunological laboratory results. In total, 119 female patients consented to and participated in this research. The participants' ages ranged from 18 to 45 years, with a mean ± SD of 33.9 ± 5.6 years.

The data were divided into nationalities; 103 patients (86.6%) were Saudi citizens, and 16 (13.4%) were non-Saudi residents. Regarding the place of residence of the patients, 69 (58.0%) were from Jeddah, 10 (8.4%) were from Makkah, 10 (8.4%) were from Taif, and 30 (25.2%) were from other cities in Saudi Arabia.

Regarding infertility types, 22 patients (18.5%) had been diagnosed with primary infertility and 97 (81.5%) with secondary infertility. Of the studied patients, 72 (60.5%) had no living children, and 47 (39.5%) had at least one living child. Recurrent abortions (more than two) were the predominant feature in 43 patients (36.1%), while 34 patients (28.6%) had no abortions. There were 67 patients (56.3%) who had received at least one ICSI, and 23 (19.3%) had received one or more IUI (Table 1).

**Table 1:** Sociodemographic characteristics and infertility background ICSI: intracytoplasmic sperm injection; IUI: intrauterine insemination.

Sociodemographic and infertility background	Number	Percentage
Age in years		
<35	61	51.3%
≥35	58	48.7%
Marital status (duration in years)		
Less than 5 years	36	30.3%
5-10 years	35	29.4%
>10 years	48	40.3%
Nationality		
Saudi	103	86.6%
Non-Saudi	16	13.4%
City		
Jeddah	69	58.0%
Makkah	10	8.4%
Taif	10	8.4%
Others	30	25.2%
Infertility type		
Primary	22	18.5%
Secondary	97	81.5%
Number of pregnancies		
None	22	18.5%
1-2 pregnancies	25	29.4%
3-4 pregnancies	22	26.9%
>4 pregnancies	30	25.2%
Number of living children		
No babies	72	60.5%
1-3 babies	42	35.3%
>3 babies	5	4.2%
Number of abortions		
No abortions	34	28.6%
1-2 abortions	42	35.3%
>2 abortions	43	36.1%
Number of stillbirths		
None	109	91.6%
Present	10	8.4%
Number of preterm		
None	111	93.3%
Present	8	6.7%
Number of ICSI		
None	52	43.7%
1-2	34	28.6%
>2	33	27.7%
Number of IUI		
None	96	80.7%
1-2	17	14.3%
>2	6	5.0%

From a total of 12 autoantibody laboratory tests, 100 patients had high levels of at least one autoimmune antibody (above the normal range), which is equivalent to 84% of the studied patients. The antithyroglobulin antibody, which was found in 42 patients (35.3%), was the commonest, followed by the antithyroid microsomal antibody in 41 patients (34.5%), while the beta 2 glycoprotein antibody IgM, found in 40 patients (33.6%), was the third common (Table 2).

**Table 2:** Autoimmunological antibodies laboratory tests according to the prevalence IgG: immunoglobulin G; IgA: immunoglobulin A; IgM: immunoglobulin M.

Autoantibodies	Normal level		High level	
	Number	Percent	Number	Percent
Antithyroglobulin antibody	77	64.7%	42	35.3%
Antithyroid microsomal antibody	78	65.5%	41	34.5%
Beta 2 glycoprotein antibody IgM	79	66.4%	40	33.6%
Antigliadin antibody IgA	87	73.1%	32	26.9%
Antinuclear antibodies	97	81.5%	22	18.5%
Anticardiolipin antibody IgM	101	84.9%	18	15.1%
Anticardiolipin antibody IgG	108	90.8%	11	9.2%
Antigliadin antibody IgG	109	91.6%	10	8.4%
Anti-tissue transglutaminase antibody IgA	111	93.3%	8	6.7%
Anti-tissue transglutaminase antibody IgG	117	98.3%	2	1.7%
Beta 2 glycoprotein antibody IgG	117	98.3%	2	1.7%
Antisperm antibody	118	99.2%	1	0.8%

There was a statistically significant association between the beta 2 glycoprotein antibody IgM and secondary infertility (p = 0.022). Moreover, the antithyroglobulin antibody was found to be increased in women over the age of 35 years, compared to younger women (p = 0.027). In addition, the number of pregnancies was statistically significantly associated with the anti-tissue transglutaminase antibody IgG and antigliadin antibody IgA (p = 0.015 and p = 0.043, respectively). Finally, the anti-tissue transglutaminase antibody IgA was statistically significantly associated with the number of ICSI cycles (p = 0.024) (Tables 3, 4).

**Table 3:** Autoantibodies correlation with sociodemographic characteristics and infertility background * P-value significant at alpha = 0.05. IgG: immunoglobulin G; IgA: immunoglobulin A; IgM: immunoglobulin M.

Autoantibodies	Age in years			Marital status (duration in years)				Infertility type		
	<35	≥35	P-value	<5	5-10	>10	P-value	Primary	Secondary	P-value
Beta 2 glycoprotein antibody IgG										
Normal	60	57	0.739	36	33	48	0.087	22	95	0.663
High	1	1		0	2	0		0	2	
Beta 2 glycoprotein antibody IgM										
Normal	37	24	0.122	22	27	30	0.274	10	69	0.022*
High	24	16		14	8	18		12	28	
Anticardiolipin antibody IgG										
Normal	55	53	0.536	32	30	46	0.261	19	89	0.330
High	6	5		4	5	2		3	8	
Anticardiolipin antibody IgM										
Normal	52	49	0.555	29	32	40	0.410	19	82	0.564
High	9	9		7	3	8		3	15	
Antithyroglobulin antibody										
Normal	45	32	0.027*	25	26	26	0.129	14	63	0.546
High	16	26		11	9	22		8	34	
Antithyroid microsomal antibody										
Normal	44	34	0.087	29	21	28	0.075	14	64	0.509
High	17	24		7	14	20		8	33	
Antinuclear antibodies										
Normal	50	47	0.541	27	27	43	0.171	19	78	0.380
High	11	11		9	8	5		3	19	
Antisperm antibody										
Normal	60	58	0.513	35	35	48	0.313	22	96	0.815
High	1	0		1	0	0		0	1	
Anti-tissue transglutaminase antibody IgA										
Normal	57	54	0.613	34	33	44	0.846	19	92	0.164
High	4	4		2	2	4		3	5	
Anti-tissue transglutaminase antibody IgG										
Normal	61	56	0.235	36	34	47	0.620	22	95	0.663
High	0	2		0	1	1		0	2	
Antigliadin antibody IgA										
Normal	47	40	0.216	27	26	34	0.897	14	73	0.197
High	14	18		9	9	14		8	24	
Antigliadin antibody IgG										
Normal	56	53	0.596	34	31	44	0.672	19	90	0.273
High	5	5		2	4	4		3	7	

**Table 4:** Autoantibodies correlation with sociodemographic characteristics and infertility background * P-value significant at alpha = 0.05. IgG: immunoglobulin G; IgA: immunoglobulin A; IgM: immunoglobulin M; IUI: intrauterine insemination; ICSI: intracytoplasmic sperm injection.

Autoantibodies	Pregnancies							Abortions				Stillbirth			ICSI				IUI			
	0	1	2	3	4	5-14	P	0	1-2	>2	P	0	≥1	P	0	1-2	>2	P	0	1-2	>2	P
Beta 2 glycoprotein antibody IgG																						
Normal	22	18	17	14	17	29	0.547	34	42	41	0.166	107	10	0.838	50	34	33	0.270	94	17	6	0.784
High	0	0	0	1	0	1		0	0	2		2	0		2	0	0		2	0	0	
Beta 2 glycoprotein antibody IgM																						
Normal	10	11	12	13	10	23	0.098	18	29	32	0.127	72	7	0.551	38	21	20	0.394	60	15	4	0.117
High	12	7	5	2	7	7		16	13	11		37	3		14	13	13		36	2	2	
Anticardiolipin antibody IgG																						
Normal	19	16	16	12	16	29	0.499	31	39	38	0.771	98	10	0.364	47	32	29	0.673	85	17	6	0.234
High	3	2	1	3	1	1		3	3	5		11	0		5	2	4		11	0	0	
Anticardiolipin antibody IgM																						
Normal	19	16	14	13	13	26	0.925	29	34	38	0.632	94	7	0.176	44	31	26	0.367	80	15	6	0.497
High	3	2	3	2	4	4		5	8	5		15	3		8	3	7		16	2	0	
Anti-thyroglobulin antibody																						
Normal	14	11	12	13	12	15	0.249	22	30	25	0.440	70	7	0.504	32	23	22	0.814	64	10	3	0.610
High	8	7	5	2	5	15		12	12	18		39	3		20	11	11		32	7	3	
Antithyroid microsomal antibody																						
Normal	14	11	12	12	11	18	0.823	21	29	28	0.800	73	5	0.228	38	21	19	0.294	64	10	4	0.820
High	8	7	5	3	6	12		13	13	15		36	5		14	13	14		32	7	2	
Antinuclear antibodies																						
Normal	19	15	12	13	14	24	0.843	30	35	32	0.280	89	8	0.586	42	28	27	0.982	79	14	4	0.630
High	3	3	5	2	3	6		4	7	11		20	2		10	6	6		17	3	2	
Antisperm antibody																						
Normal	22	18	17	15	16	30	0.301	34	42	42	0.410	108	10	0.916	51	34	33	0.522	95	17	6	0.886
High	0	0	0	0	1	0		0	0	1		1	0		1	0	0		1	0	0	
Anti-tissue transglutaminase antibody IgA																						
Normal	19	17	16	13	17	29	0.473	31	38	42	0.352	102	9	0.516	52	29	30	0.024*	89	17	5	0.329
High	3	1	1	2	0	1		3	4	1		7	1		0	5	3		7	0	1	
Anti-tissue transglutaminase antibody IgG																						
Normal	22	18	17	13	17	30	0.015*	34	41	42	0.666	107	10	0.838	51	33	33	0.635	94	17	6	0.784
High	0	0	0	2	0	0		0	1	1		2	0		1	1	0		2	0	0	
Antigliadin antibody IgA																						
Normal	14	12	14	9	10	28	0.043*	21	31	35	0.154	82	5	0.093	43	21	23	0.088	70	13	4	0.893
High	8	6	3	6	7	2		13	11	8		27	5		9	13	10		26	4	2	
Antigliadin antibody IgG																						
Normal	19	16	17	13	16	28	0.662	30	38	41	0.508	101	8	0.199	49	31	29	0.586	87	17	5	0.331
High	3	2	0	2	1	2		4	4	2		8	2		3	3	4		9	0	1	

## Discussion

Unexplained infertility, mainly with recurrent pregnancy losses and implantation failures, constitutes a particularly challenging and devastating topic in reproductive medicine and places a significant emotional burden on the couples involved.

Recent research has shown that a successful pregnancy includes an immunologically unique phase of temporary tolerance mechanism toward the implanting fetus, to allow implantation and the subsequent development of the pregnancy without rejection [16]. Various recent studies have suggested that an overactive immune system in some females may increase the difficulty in conceiving or increase the risk of abortion [7]. This suggests that there could be a better chance of success by investigating the immune system and trying immunomodulating therapies.

In this cross-sectional study, 12 different auto-immunological antibodies laboratory tests were done on 119 female patients with unexplained infertility. The overall prevalence of at least one positive test result for autoimmune antibodies was 84%, which corresponds to the majority of the studied group. Two previous similar studies done in France explored fewer autoantibodies and found that the prevalence of any autoantibodies was 32-33% [17,18]. This might reflect the complexity of the cases referred to an immunology expert and the need for a broader selection of and more specific group of autoantibodies.

In this study, the most predominant high level of autoantibodies was antithyroid (antithyroglobulin and antithyroid microsomal) antibodies, which were found in one-third of the studied group. Interestingly, the levels of antithyroglobulin antibodies increased with an increase in the women’s ages. In two previous studies on unexplained infertility, 29% of the cases were found to have antithyroid peroxidase antibodies [18,19]. The effects of thyroid antibodies on infertility, IVF outcome, and ongoing pregnancy are controversial, but, if these antibodies coexist with hyperthyroidism or subclinical hypothyroidism, they could impair fertility [11,20].

In this study, antiphospholipid antibodies were the second most predominant autoantibodies found in high levels in almost one-third of the studied group, particularly B2 glycoprotein, and more than anti-cardiolipin antibodies, which were reported in previous similar studies to be higher than 22-23% [19,21]. Interestingly, the studied group showed a significant relationship between B2 glycoprotein and secondary infertility. Although some studies have shown that antiphospholipid antibodies do not correlate with pregnancy and IVF outcomes, other studies have shown a lower clinical pregnancy rate [11,20].

The prevalence of high levels of antinuclear antibodies found in this study was 18.5%, which is close to what was found in a recent large meta-analysis that found a 22% prevalence within the recurrent pregnancy loss group [8]. Moreover, a systemic review has shown that the presence of anti-nuclear antibodies was associated with fewer clinical pregnancies and higher miscarriage rates after IVF [8,20]. More specifically, celiac antibodies such as the anti-tissue transglutaminase IgA were found in only 6.4% of the studied patients, which correlates with previous studies that found 2-8% [10]. High levels of antigliadin IgA, a less specific celiac antibody, were found in almost a quarter of the studied patients. In contrast, a recent meta-analysis conducted in 2021 has shown that celiac disease is not more common in infertile women than in the general population, which is in contrast to what was found in two previous meta-analyses [22]. However, in this study, high levels of anti-tissue transglutaminase IgG and anti-gliadin IgA antibodies were associated with an increased number of both natural and assisted pregnancies, and anti-tissue transglutaminase antibody IgA was associated with an increased number of ICSI cycle failures.

However, some autoantibodies were not common in this studied group such as beta 2 glycoprotein antibody IgG, anti-tissue transglutaminase IgG, and antisperm antibodies. Hence, the performance of these autoantibody tests may not be regular in patients with unexplained infertility, unless it is for further research purposes.

Determining common immunological irregularities may aid in establishing a guideline as to when these autoimmunological markers should be used and which types to consider to widen the perspective of the physician to be able to establish the relationship between the immune system and unexplained infertility in females.

It should be noted that this research was subject to several limitations that included using convenient sampling from an allergy immunology clinic, missing files, a small sample size, and a non-randomized study.

## Conclusions

In conclusion, this cross-sectional study has revealed that raised level of at least one autoantibody was a common finding in this convenient sample of females with unexplained infertility. Among those, antithyroid, antiphospholipid, antigliadin, and antinuclear autoantibodies were the commonest autoantibodies. Encouraging the identification and isolation of specific autoimmunological pathways of maternal immune responses to the fetus may benefit the clinical investigation approach and the management of females with unexplained infertility. Further large-scale and well-designed randomized controlled clinical studies that examine the benefits of autoimmunological evaluations in females with infertility are necessary before more detailed recommendations can be created.
